# Obituary

**Published:** 1900-09-15

**Authors:** 


### OBITUARY.

### HENRY H. BURCHARD, M.D., D.D.S.

Dr. Burchard died June 25, 1900, at Redlands, Cal.

Dr. Burchard was born in Philadelphia, September 20, 1862, and finished his preliminary education at the Philadelphia High School. In 1879 he entered the engineer class of the United States navy, but left that to take up the study of dentistry.

In 1885 he graduated as Doctor of Dental Surgery at the Philadelphia Dental College. In 1888 he graduated from the Jefferson Medical College of Philadelphia and began the practice of medicine, which he abandoned after three years' experience and returned to the practice of dentistry, which he continued until failing health obliged him to resort to teaching and literary work.

His contributions to dental periodical literature have been varied and important. In 1896 he was elected to the Chair of Pathology and Therapeutics in the Philadelphia Dental College, and continued in this service until obliged to resort to a more congenial climate.

The principal work of his later years, and which contributed largely to the further undermining of an already enfeebled organization, was the editing and the contribution of eight chapters to Essig's "Prosthetic Dentistry." He also wrote several chapters and made one hundred drawings for Kirk's "Operative Dentistry." This laborious work was immediately followed by the preparation of his own book of "Dental Pathology, Therapeutics and Pharmacology," issued in 1898. He had previously prepared a "Compend on Dental Pathology and Therapeutics." In addition to the before-mentioned works, he revised the dental definitions for Duane's Dictionary, and also performed the same service for Gould's Dictionary. He wrote sections on Plastics, Vol. IV., "American System of Dentistry," and revised dental portions in the thirteenth edition of Gray's "Anatomy," besides contributing extensively to the illustrations. He was also one of the assistant editors in the science department of Catching's "Compendium." He was a member of several dental societies, also of the Franklin Institute of Philadelphia and the Biological Section of the Academy of Natural Sciences of the same city.

This brief *resume* of his work furnishes but a limited idea of his untiring industry. He seemed to feel that he must not only work while the day lasted, but far into the nights, and, while he enjoyed social life, he allowed himself but little time for its pleasures.

While he lived only thirty-eight years, he accomplished much more than most men of three score years and ten.—*International Dental Journal.*

